# Association of Odor Identification Ability With Amyloid-β and Tau Burden: A Systematic Review and Meta-Analysis

**DOI:** 10.3389/fnins.2020.586330

**Published:** 2020-11-26

**Authors:** Lihui Tu, Xiaozhen Lv, Zili Fan, Ming Zhang, Huali Wang, Xin Yu

**Affiliations:** ^1^Dementia Care and Research Center, Clinical Research Division, Peking University Institute of Mental Health (Sixth Hospital), Beijing, China; ^2^Beijing Dementia Key Lab, National Clinical Research Center for Mental Disorders, Key Laboratory of Mental Health, Ministry of Health Peking University, Beijing, China; ^3^Department of Psychiatry, The Third Affiliated Hospital of Sun Yat-sen University, Guangzhou, China

**Keywords:** olfaction, Alzheimer's disease, amyloid-β, tau, positron emission tomography, cerebrospinal fluid

## Abstract

**Background:** The associations between olfactory identification (OI) ability and the Alzheimer's disease biomarkers were not clear.

**Objective:** This meta-analysis aimed to examine the associations between OI and Aβ and tau burden.

**Methods:** Electronic databases (PubMed, Embase, PsycINFO, and Google Scholar) were searched until June 2019 to identify studies that reported correlation coefficients or regression coefficients between OI and Aβ or tau levels measured by positron emission tomography (PET) or cerebrospinal fluid (CSF). Pooled Pearson correlation coefficients were computed for the PET imaging and CSF biomarkers, with subgroup analysis for subjects classified into different groups.

**Results:** Nine studies met the inclusion criteria. Of these, five studies (*N* = 494) involved Aβ PET, one involved tau PET (*N* = 26), and four involved CSF Aβ or tau (*N* = 345). OI was negatively associated with Aβ PET in the mixed (*r* = −0.25, *P* = 0.008) and cognitively normal groups (*r* = −0.15, *P* = 0.004) but not in the mild cognitive impairment group. A similar association with CSF total tau in the mixed group was also observed. No association was found between OI and CSF phosphorylated tau or Aβ_42_ in the subgroup analysis of the CSF biomarkers. Due to a lack of data, no pooled *r* value could be computed for the association between the OI and tau PET.

**Conclusion:** The associations between OI ability and Aβ and CSF tau burden in older adults are negligible. While current evidence does not support the association, further studies using PET tau imaging are warranted.

## Introduction

Amyloid-β (Aβ) aggregates and tau neurofibrillary tangles are known as the two neuropathological hallmarks of Alzheimer's disease (AD) (Villemagne et al., 2018). The importance of the two biomarkers, whether for clinical or research use, is obvious in the biologically oriented effort to tackle the worldwide AD issue (Jansen et al., 2015; Alzheimer's Association, 2020). This is especially true when the National Institute on Aging and Alzheimer's Association (NIA-AA) proposed the Aβ/tau/neurodegeneration (AT(N)) classification system to update the diagnostic criteria for AD recommended in 2011(Jack et al., 2018). This biomarker-driven research framework has shown potential to improve the predictive accuracy for memory decline among non-demented elderly individuals and thereby provide prognostic values for clinical change and progression (Jack et al., 2019; Yu et al., 2019).

In the evolving biomarker research field of AD, olfactory function has been frequently seen in studies related to neurodegenerative diseases (Marin et al., 2018; Dintica et al., 2019; Tu et al., 2020) since its first association with dementia was claimed in 1974 (Waldton, 1974). Impaired olfaction, olfactory identification (OI) ability in particular, has been reported in AD and prodromal AD ailments, such as mild cognitive impairment (MCI) (Roalf et al., 2017; Marin et al., 2018; Jung et al., 2019). Moreover, several lines of evidence have indicated that OI impairment is valuable in predicting cognitive decline in cognitively intact participants and progression from MCI to AD dementia, and it might suggest neurodegeneration in the brain among non-demented older adults (Devanand et al., 2015; Roberts et al., 2016; Dintica et al., 2019). OI, the ability to identify and name specific odorants, is therefore considered a potential early biomarker of cognitive decline and AD dementia.

Due to the well-known disadvantages of current standard measures of AD biomarkers, namely, their expensive cost and invasiveness, a convenient, inexpensive, and easily accessible test that predicts amyloid and tau status would therefore reduce the burden and cost of clinical AD trials. Studies have attempted to identify correlations of OI ability with amyloid and tau burden both *in vitro* and *in vivo*. Postmortem studies (Kovacs et al., 1999; Attems et al., 2005; Attems and Jellinger, 2006; Wilson et al., 2007, 2009; Franks et al., 2015) have linked OI ability with the pathologic manifestations of AD, Aβ, and neurofibrillary tangles. Studies involving *in vivo* positron emission tomography (PET) imaging scans or cerebrospinal fluid (CSF) measures also showed interesting results. But the results are inconsistent and inconclusive; the aim of this systematic review was therefore to provide a comprehensive overview of OI ability associated with the Aβ and tau burden in older adults.

## Methods

### Selection Criteria

This systematic review was conducted according to the PRISMA guidelines (Moher et al., 2015) and followed a predetermined protocol (PROSPERO No. CRD42019138642). The selection criteria of the studies were as follows: (1) reported associations between OI test scores and Aβ or tau levels (measured via PET imaging or CSF); (2) evaluated OI by common smell tests, such as the University of Pennsylvania Smell Identification Test (UPSIT), the “Sniffin' Sticks” OI test, and the Odor Stick Identification Test for Japanese (OSIT-J); (3) included older adults (mean age of sample ≥ 60 years); and (4) made data available in the publication or via contact with the authors to allow computation of correlation coefficients.

### Search Strategy

Systematic electronic databases (PubMed, Embase, PsycINFO, and Google Scholar) were searched for articles published in English from their inception until June 2019 with the following search items: “olfaction” OR “smell” OR “odor” OR “olfactory” AND “amyloid” OR “tau” OR “cerebrospinal fluid” OR “positron emission tomography.” Filters were applied to limit searches to human studies in the English language. The reference lists and similar articles of the eligible publications were searched manually for additional studies.

### Amyloid and Tau Assessment

PET amyloid imaging agents included Pittsburgh Compound B (PIB), florbetapir, and florbetaben. PET tau imaging agents included tau-specific ligands, such as flortaucipir. Estimates of amyloid and tau binding in PET studies were used from the global cortex or cortical regions via standardized uptake value ratios (SUVRs) or distribution volume ratios (DVRs). The CSF method included measurement of Aβ 1-_42_ (Aβ_42_), *p*-tau_181_, and *t*-tau levels.

### Odor Identification Test

OI was tested with the UPSIT, Sniffin' Sticks, OSIT-J, or other commercially available tests. The shorter versions of the UPSIT and Sniffin' Sticks test were also eligible. Homemade tests and tests that also assessed other olfactory functions (e.g., olfactory threshold or discrimination) were excluded.

### Data Extraction and Study Quality Assessment

Data were independently extracted by two investigators (LT and XL) from cross-sectional cohort studies and baseline measurements of longitudinal studies with clinical follow-up. For the studies with different groups of subjects, i.e., cognitively normal (CN), MCI, AD, and mixed (whole sample) groups, the correlation coefficient values were extracted separately for the subgroup comparisons when data were available. The following information was extracted from each included study: the sample size, the study design, the country and cohort name, the methodology used to measure AD biomarkers, the odor tests, the sample demographic characteristics, and the bivariate correlations (or related statistical information) between the AD biomarkers and the OI score. The methodologic quality of each included study was assessed using the Quality Assessment of Diagnostic Accuracy Studies (QUADAS-2) tool (Whiting et al., 2011).

### Effect Size Computation and Statistical Analysis

The cross-sectional associations between OI test scores and Aβ or tau levels were evaluated using Pearson correlation coefficients (*r*). A preadjusted *r* was used in all studies when available. In cases where *r* values were not reported, they were calculated from the scatter plot graph of the OI score vs. Aβ or tau levels. Two studies (Körtvélyessy et al., 2015; Risacher et al., 2017) reported a non-significant association between the OI score and Aβ or tau levels, but no data were available. In this case, *z* = 0.00 was assigned as a conservative estimate. The other three studies reported an unstandardized regression coefficient (*b*) (Growdon et al., 2015; Reijs et al., 2017; Vassilaki et al., 2017), with which we calculated *r* according to previous methods (Kim et al., 2019). Specifically, the following formulas were used:

(Estimated *r*)^2^ = *t*^2^ / (*t*^2^ + *n* – 2)

*t* = *b*/the standard error of *b*

Estimated *r* × *b* ≥ 0.

We used the Comprehensive Meta-Analysis (CMA), version 3 software (Biostat, NJ) to compute the *r* values and calculate the pooled mean *r* values for the PET imaging and CSF biomarkers, with subgroup analysis for subjects classified into different groups. As random-effects models incorporate between-study heterogeneity and give wider (i.e., more conservative) confidence intervals (CIs) when heterogeneity is suspected, all analyses and plots were reported using a random-effects model. The presence of publication bias with a funnel plot was not assessed because very few studies were included in our meta-analysis (Sterne et al., 2011). To examine between-study heterogeneity, in addition to Cochran's *Q* (to determine whether the between-study variability was greater than the sampling error) and τ (to quantify the between-study variance of the true effect sizes), the *I*^2^ statistic was also used.

*Post hoc* subgroup analyses were conducted to determine the source of the heterogeneity when statistically significant heterogeneity was identified. The analyses examined the degree to which heterogeneity resulted from variance due to moderators such as (a) the type of OI test (e.g., UPSIT edition), (b) the PET imaging method (PIB or non-PIB) and the measurements (SUVR or DVR), (c) the code of the SUVR/DVR (continuous or categorical), (d) adjustments for covariates (e.g., age, sex) or lack thereof, (e) the sample size, and (f) the method *r* was obtained.

## Results

### Description of Studies

**Figure 1:**
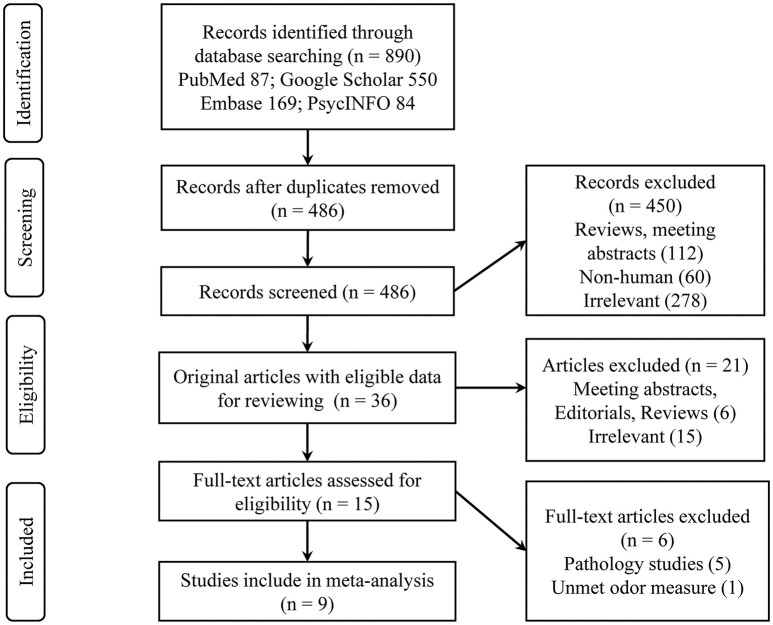
Representation of the search strategy.

A total of nine eligible studies were included in the final systematic review and meta-analysis (see Figure 1 for flowchart), with five studies pertaining to Aβ PET and four about CSF Aβ or tau. The characteristics of the included studies are shown in Table 1. The selected studies were published between 2010 and 2018. Seven studies were cross-sectional, and two were longitudinal. The median number of subjects per study was 93 (range, 22–215), with a total number of 839 (56% female) subjects. Three studies with a relatively large sample (over 100) focused on CN older adults, two of which utilized the PET imaging method (Growdon et al., 2015; Vassilaki et al., 2017). For the PET method, the included total sample sizes of CN, MCI, and AD individuals were 392, 75, and 20, respectively, with seven subjective cognitive decline (regarded as CN in our analysis) individuals. The sample sizes for the studies using the CSF method were 161, 63, and 95 individuals, respectively, with 26 non-AD dementia patients. The mean sample age was 71.8 years (SD = 6.8).

**Table 1:** Characteristics of the included studies.

**References**	**Design (follow-up)**	**Country**	***N***	**Cohort**	**Mean age [year (SD)]**	**Sex, female (%)**	**Olfactory test**	**AD biomarker**	**Classification**	**Statistical analysis**
Bahar-Fuchs et al. (2010)	XS	Australia	63	Longitudinal PiB PET project at Austin Health	73.6 (8.2)	58.6	UPSIT (6-item)	PET: Aβ (^11^C PIB)	CN, MCI, AD	Pearson correlation
Growdon et al. (2015)	XS	USA	215	Harvard Aging Brain Study	73.9 (5.9)	59.1	UPSIT	PET: Aβ (^11^C PIB)	CN	Multiple linear regression
Kreisl et al. (2018)	LT (1 year)	USA	71	Longitudinal observational study of AD biomarkers	68.5 (7.6)	49.0	UPSIT	PET: Aβ (^11^C PIB)	CN, MCI	Pearson correlation
Risacher et al. (2017)	XS	USA	26	Indiana Alzheimer Disease Center	70.4 (8.8)	63.4	UPSIT	PET: Aβ (^18^F-florbetapir/florbetaben); Tau (^18^F-flortaucipir)	CN, SCD, MCI	Pearson correlation
Vassilaki et al. (2017)	XS	USA	119	Mayo Clinic Study of Aging	79.2 (–)	48.5	UPSIT (12-item)	PET: Aβ (^11^C PIB), ^18^F-FDG-PET	CN	Multiple linear regression
Körtvélyessy et al. (2015)	XS	Germany	22	Memory Clinic, University of Magdeburg	72.7 (6.9)	66.7	Sniffin (12-item)	CSF: Aβ_42_, *p*-tau, *t*-tau	AD	Pearson correlation
Kouzuki et al. (2018)	XS	Japan	71	Faculty of Medicine, Tottori University	78.3 (1.1)	43.8	OSIT-J	CSF: Aβ_42_, *p*-tau	CN, MCI, AD	Pearson correlation
Lafaille-Magnan et al. (2017)	XS	Canada	100	The PREVENT-AD cohort	62 (6)	70.0	UPSIT	CSF: Aβ_42_, *p*-tau, *t*-tau	CN	Multiple linear regression
Reijs et al. (2017)	LT (3 years)	Netherlands	152	The EDAR study	67.4 (9.5)	47.2	UPSIT (12-item)	CSF: Aβ_42_, *t*-tau	CN, MCI, AD, non-AD dementia	Multiple linear regression

*XS, cross sectional; LT, longitudinal; UPSIT, University of Pennsylvania Smell Identification Test; OSIT-J, Odor Stick Identification Test for Japanese; PET, positron emission tomography; ^11^C PIB, ^11^C-Pittsburgh compound B; Aβ, amyloid β; CSF, cerebrospinal fluid; Aβ_42_, amyloid-β_42_; t-tau, total tau; p-tau, phosphorylated tau; ^18^F-FDG, ^18^fluorodeoxyglucose; CN, cognitively normal; SCD, subjective cognitive decline; MCI, mild cognitive impairment; AD, Alzheimer's disease*.

Demographic variables such as age, sex, and education were adjusted statistically in a few studies. The inclusion criteria for individuals with MCI were mostly according to Petersen's criteria, while for AD, it was the NINCDS-ADRDA criteria. The methodologic quality assessment showed that only the patient selection domain had the high risk of bias, mostly due to inappropriate exclusions (Table 2).

**Table 2:** Risk of bias and applicability concern summary: review authors' judgements about each domain for included studies, individually.

**Study**	**Risk of bias**				**Applicability concerns**		
	**Patient selection**	**Index test**	**Reference standard**	**Flow and timing**	**Patient selection**	**Index test**	**Reference standard**
1. Bahar-Fuchs	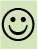	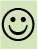	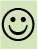	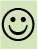	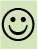	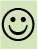	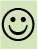
2. Growdon	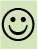	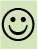	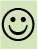	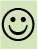	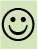	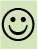	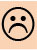
3. Kreisl	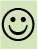	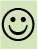	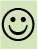	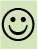	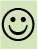	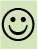	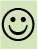
4. Risacher	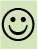	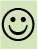	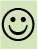	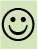	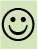	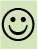	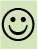
5. Vassilaki	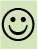	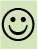	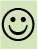	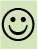	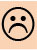	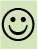	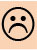
6. Körtvélyessy	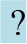	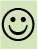	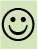	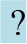	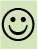	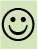	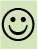
7. Kouzuki	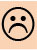	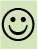	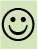	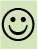	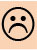	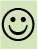	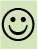
8. Lafaille-Magnan	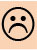	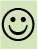	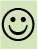	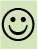	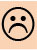	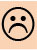	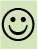
9. Reijs	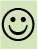	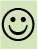	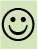	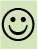	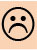	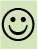	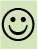

*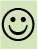, low risk; 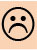, high risk; 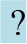, unclear risk*.

### AD Biomarkers

Five studies applied Aβ PET imaging [four used ^11^C PIB (Bahar-Fuchs et al., 2010; Growdon et al., 2015; Vassilaki et al., 2017; Kreisl et al., 2018); one used ^18^F-florbetapir/florbetaben (Risacher et al., 2017)], and only one study utilized tau PET imaging [^18^F-flortaucipir (Risacher et al., 2017)]. For the majority of studies, the PET SUVR was used as a continuous variable to explore the association between the Aβ burden and OI score. Four studies measured CSF levels, two of which examined Aβ_42_, *p*-tau, and *t*-tau (Körtvélyessy et al., 2015; Lafaille-Magnan et al., 2017), whereas one study measured Aβ_42_ and *p*-tau (Kouzuki et al., 2018), and the other one measured Aβ_42_ and *t*-tau (Reijs et al., 2017).

### Odor Identification Score

The OI score was obtained mostly (seven studies) from the UPSIT, with 40 odors used by four studies and a short edition (6~12 odors) used by three studies. The other two studies used either Sniffin' Sticks (Körtvélyessy et al., 2015) or the OSIT-J (Kouzuki et al., 2018). The raw OI scores were reported by all studies except one (Lafaille-Magnan et al., 2017), which used a transformed UPSIT error score. In this case, the raw score was calculated from the scatter plot graph. One study (Dhilla Albers et al., 2016) was excluded because a homemade olfactory screening with mixed evaluation was adopted.

### Correlation Between the Odor Identification Score and PET Imaging

**Figure 2:**
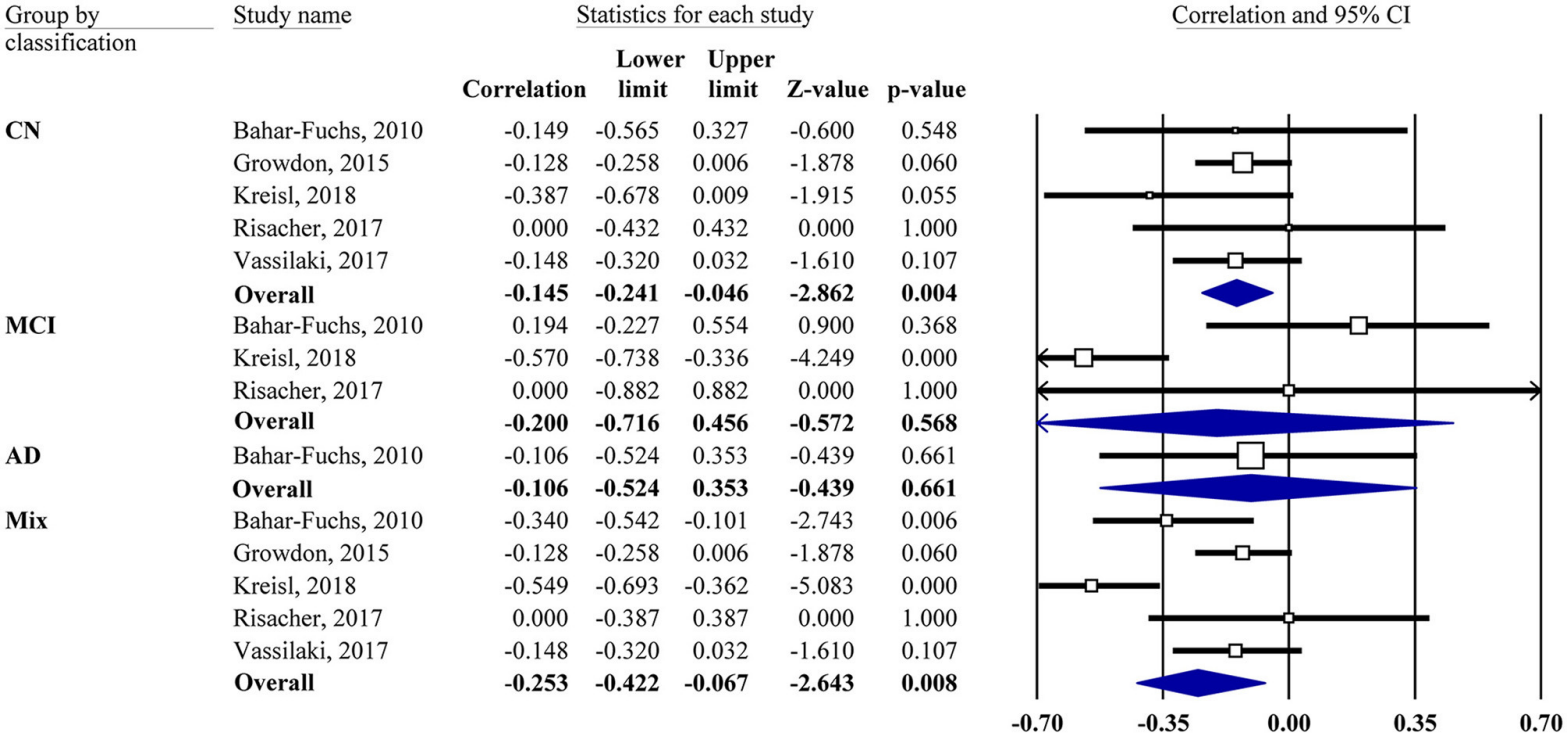
Forest plot summarizing the correlations between the odor identification score and amyloid PET imaging data and their 95% confidence intervals for different groups. (Squares represent study weighting due to sample size, and the diamond represents the weighted mean effect size estimated in a random-effects model. CN, cognitively normal; MCI, mild cognitive impairment; AD, Alzheimer's disease).

Meta-analyses were based on the correlation between the OI score and the PET SUVR or DVR. For two studies (Growdon et al., 2015; Vassilaki et al., 2017), the *r* values were calculated based on the regression coefficients. For correlations of subgroup subjects (CN, MCI, and AD), the *r* values were obtained via the authors for one study (Kreisl et al., 2018) and calculated from the scatter plot graph for another study (Bahar-Fuchs et al., 2010). One study (Risacher et al., 2017) reported a non-significant association without available data, in which *z* = 0.00 was assigned accordingly. Our results showed that the OI score was negatively associated with Aβ PET SUVR or DVR in the mixed group (*r* = −0.25, 95% CI [−0.42, −0.07], *P* = 0.008; Figure 2).

Subgroup analysis also showed a negative association in the CN group (*r* = −0.15, 95% CI [−0.24, −0.05], *P* = 0.004). However, the MCI group showed no correlation (*r* = −0.2, 95% CI [−0.72, 0.46], *P* = 0.568). Only one study had an AD group, which also showed no correlation. The combination of MCI and AD together did not change the result (data not shown). The association between the OI score and tau PET imaging was reported by one study (Risacher et al., 2017), showing that the tau level in the mean temporal lobe was negatively associated with the preadjusted UPSIT total score (*r* = −0.45, *P* < 0.05).

### Correlation Between the Odor Identification Score and CSF Biomarkers

Meta-analyses were based on the correlation between the OI score and CSF biomarker levels. For two studies, the *r* values were calculated based on the regression coefficients (Reijs et al., 2017) or from the scatter plot graph (Lafaille-Magnan et al., 2017). One study (Körtvélyessy et al., 2015) reported a non-significant association without available data, in which *z* = 0.00 was assigned accordingly. MCI and AD were analyzed together given the limited data.

**Figure 3:**
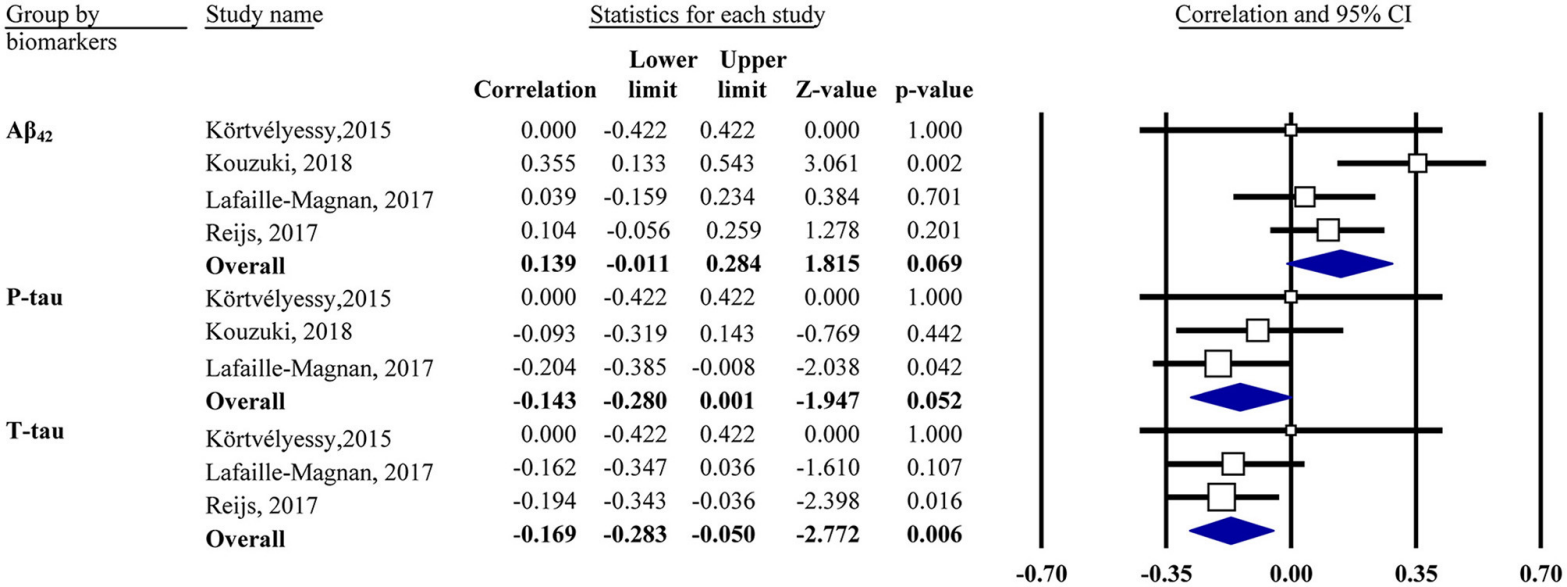
Forest plot summarizing the correlations between the odor identification score and cerebrospinal fluid biomarker levels and their 95% confidence intervals in the mixed group. Squares represent study weighting due to sample size, and the diamond represents the weighted mean effect size estimated in a random-effects model. Aβ_42_, amyloid-β_42_; *t*-tau, total tau; *p*-tau, phosphorylated tau.

Our analysis showed that the OI score was negatively associated with CSF *t*-tau in the mixed group (*r* = −0.17, 95% CI [−0.28, −0.05], *P* = 0.006; Figure 3). The same was true for CSF *p*-tau (*r* = −0.14, 95% CI [−0.28, 0.001], *P* = 0.052) and Aβ_42_ (*r* = 0.14, 95% CI [−0.01, 0.28], *P* = 0.069) in the mixed group but only with a marginal association. Subgroup analysis showed no association in the CN or MCI/AD group for CSF Aβ_42_ or *t*-tau (data not shown).

### Moderator Effects

The present data showed a statistically significant heterogeneous between-study variability for the PET imaging method in the mix (*Q* = 15.2, *P* < 0.01, τ^2^ = 0.033, *I*^2^ = 73.68%) and MCI groups (*Q* = 10.3, *P* < 0.01, τ^2^ = 0.267, *I*^2^ = 80.64%). *Post hoc* subgroup analyses indicated that the pooled *r* values in the mixed group remained statistically significant when the moderator variables were adjusted (Table 3).

**Table 3:** Pooled Pearson's *r* values and 95% confidence intervals (CIs) adjusted for moderator variables (for Aβ PET only).

**Moderator variables**		**MCI**		**Mix**	
		***r* [95% CI]**	***I*^**2**^**	***r* [95% CI]**	***I*^**2**^**
Odor test	UPSIT-L	−0.55 [−0.72, −0.32]***	0	−0.26 [−0.56, 0.1]	85
	UPSIT-S	Bahar-Fuchs et al. (2010)+		−0.23 [−0.4, −0.04]*	40
PET method	PIB	−0.24 [−0.79, 0.53]	90	−0.29 [−0.47, −0.09]**	79
	Non-PIB	Risacher et al. (2017)		Risacher et al. (2017)	
PET measure	SUVR	−0.2 [−0.72, 0.46]	81	−0.35 [−0.57, −0.09]*	79
	DVR	–		Growdon et al. (2015)	
Amyloid analysis	Continuous	−0.2 [−0.72, 0.46]	81	−0.35 [−0.59, −0.05]*	71
	Categorical	–		−0.14 [−0.24, −0.03]*	0
Sample size	>100	–		−0.14 [−0.24, −0.03]*	0
	<100	−0.2 [−0.72, 0.46]	81	−0.35 [−0.59, −0.05]*	71
Method of obtaining *r*	Reported	Kreisl et al. (2018)		−0.45 [−0.63, −0.23]***	54
	Estimated	0.18 [−0.23, 0.53]	0	−0.13 [−0.23, −0.02]*	0

*The results for adjusted covariates equaled those of the odor test*.

*PET, positron emission tomography; UPSIT, University of Pennsylvania Smell Identification Test, -L = 40 items, -S = 6~12 items; PIB, Pittsburgh Compound B; SUVR, standardized uptake value ratio; DVR, distribution volume ratio; MCI, mild cognitive impairment*.

*“+” references*.

*P < 0.05; **P < 0.01; ***P < 0.001.

A significant negative correlation (*r* = −0.55, 95% CI [−0.72, −0.32], *P* < 0.001) for the MCI group was observed when these accounted for data from studies that had utilized the 40-item UPSIT, although this only pertained to two studies. Table 3 provides a summary of the meta-analysis by potential moderator variables of the association between the OI score and PET imaging.

## Discussion

This meta-analysis explored the relationships between OI ability and the cerebral measures of amyloid and tau deposition via PET imaging or CSF evaluation. Our main finding was that Aβ (measured by PET) and *t*-tau (measured by CSF evaluation) depositions were only weakly associated with OI scores across a mixed population (CN, MCI, and AD) of older adults. A weak association was also observed in the CN group between the Aβ deposition (measured by PET) and OI score. As all the pooled absolute *r* values were <0.3, the correlation could be considered negligible. There were no associations between the MCI and AD groups or between CSF Aβ_42_ and *t*-tau. In addition, no pooled *r* value could be computed for the association between the OI score and tau PET due to the lack of data. These findings suggest that the associations between OI ability and Aβ and CSF tau burden in older adults are negligible. OI ability is believed to be linked with the pathologic manifestations of AD in postmortem studies (Kovacs et al., 1999; Attems et al., 2005; Attems and Jellinger, 2006; Wilson et al., 2007, 2009; Franks et al., 2015). Among the AD pathologic changes associated with OI, neurofibrillary tangles were particularly noted, especially in olfactory bulb (OB) (Kovacs et al., 1999; Attems et al., 2005; Attems and Jellinger, 2006) and central olfactory regions (entorhinal cortex and CA1/subiculum area of the hippocampus) (Wilson et al., 2007). This was in line with the tau PET imaging study (Risacher et al., 2017) included in our analysis, which indicated a low negative correlation between the tau and OI score in the mean temporal lobe. Of note, this was in response to the idea endorsed by Kametani et al. suggesting that it is tau contributed to the development and progression of AD, not Aβ (Kametani and Hasegawa, 2018). Nevertheless, the meta-analysis of *in vivo* CSF *t*-tau, not *p*-tau, yielded only a very weak association, which was considered negligible. The inconsistency of the two findings might be explained by the following aspects: the histopathological study contained a relatively small sample size with advanced age and a span of several years between olfactory testing and death. More importantly, *in vitro* neuropathological studies focused mainly on specific olfactory-related regions, while *in vivo* CSF studies could not take the same approach. However, the association with OI for the Aβ burden on autopsy appeared non-significant, which supported our meta-analysis findings, particularly the results regarding CSF Aβ, despite a low negative correlation that could be observed in the mixed group with moderator variables adjusted for the Aβ PET imaging.

The mixed group, in general, showed a negligible correlation between OI ability and the Aβ and CSF tau burden. However, the MCI and AD groups, both of which are associated with OI impairment and inclined to be associated with Aβ or tau burden (Jansen et al., 2015; Roalf et al., 2017; Villemagne et al., 2018; Jung et al., 2019), showed no correlation between the two at all among the different biomarker classifications. The correlation was observed in the MCI group for Aβ PET when only the studies that applied the 40-item UPSIT were included, which was largely due to one study alone (Kreisl et al., 2018). Although part of the irrelevant finding may be related to the relatively small size of the sample within the included studies, especially for the AD group with Aβ PET imaging (*n* = 20), we speculate that OI ability is not specific to the underlying AD pathophysiology, especially Aβ. On the other hand, the CN group is usually characterized by intact OI ability and a lack of Aβ or tau burden, making the correlation between the two likely. In fact, Kreisl et al. (2018) attributed the correlation between OI ability and PIB SUVR largely to the subjects with high OI scores and low PIB binding in their study. Nevertheless, our results did not show any correlations in the CN group, with a negligible association for Aβ PET imaging. This result again suggests that OI ability is non-specific for the underlying Aβ burden.

The weak association between OI ability and the cerebral measures of amyloid and CSF tau levels might be explained by several possibilities. First, like the notion mentioned above, OI impairment may not be specific to AD. It is known that impaired OI is associated with normal aging, and some age-related changes in olfactory function may relate to factors irrelevant to AD pathophysiology (e.g., deterioration of the olfactory epithelium and ossification of the cribriform plate) (Doty et al., 1984; Doty and Kamath, 2014). In addition, many neuropsychiatric disorders, such as Parkinson's disease (Hoyles and Sharma, 2013), dementia with Lewy bodies (Mahlknecht et al., 2015), and schizophrenia (Kamath et al., 2019), are reported to be associated with OI impairment. In addition to AD pathologic alterations, alpha-synucleinopathy of Parkinson's disease and dementia with Lewy bodies in the cortical brain and olfactory-related regions are also observed and are supported by convincing evidence (Wilson et al., 2007; Arnold et al., 2010; Nag et al., 2019).

Second, the limitations of the Aβ burden itself and its current measures are worth noting. The Aβ burden is not conclusive in determining the risk of AD or cognitive impairment. The prevalence of incidental Aβ positivity increases with age, and approximately a quarter of CN elders are amyloid-positive on PET scans or CSF evaluations (Jansen et al., 2015); the so-called asymptomatic cerebral amyloidosis stage may make the correlation between cerebral amyloid and OI impairment fruitless because the latter occurs predominantly in MCI and AD but not CN (Jansen et al., 2015; Jung et al., 2019). It is also worth noting that Aβ burden reaches a plateau early in the disease process or even in the preclinical phase of the AD (Ingelsson et al., 2004; Serrano-Pozo et al., 2011); hence, it is not the most appropriate to correlate OI with Aβ in AD, especially in late stage. Furthermore, a more toxic soluble or oligomeric form of Aβ (Walsh et al., 2002; Shankar et al., 2008), which is considered critical in the AD pathological cascade but has not been measured in the included studies, may potentially correlate with OI ability more directly (Bahar-Fuchs et al., 2010).

Third, the same concept may apply to CSF *t*-tau and *p*-tau, which are often used to stage preclinical AD and are viewed as biomarkers of a “disease state,” despite potentially correlating with OI ability (Mattsson et al., 2017; Lian et al., 2019). It was suggested that CSF P-tau levels might vary among AD and occur before measurable cognitive decline, which also makes the correlation difficult (Leuzy et al., 2019; Meyer et al., 2020). However, there are advantages to associating PET tau, viewed as a biomarker of a “disease stage,” with dementia status and cognitive decline (Brier et al., 2016; Mattsson et al., 2017). Risacher et al. (2017) stated that tau deposition significantly correlated with OI ability, on the condition that Aβ was positive. This, to a certain extent, corresponds with the definition of AD under the AT(N) scheme (Jack et al., 2018), which requires that both Aβ and tau are positive. Therefore, correlating OI ability with PET tau will be promising and plausible, especially when focusing on Aβ-positive individuals.

Finally, the included PET studies focused mainly on composite gray matter from frontal, parietal, lateral temporal cortex, and other regions of interest (Growdon et al., 2015; Vassilaki et al., 2017; Kreisl et al., 2018) but olfactory structure, while an olfactory region-targeted evaluation approach may strengthen the exploratory association. A number of neuroimaging studies indicate the association between structural and functional degeneration of distinct brain regions and olfactory impairment, mainly to the hippocampus and the primary olfactory cortex (Thomann et al., 2009; Growdon et al., 2015; Vasavada et al., 2015, 2017; Risacher et al., 2017; Vassilaki et al., 2017; Wu et al., 2019). In addtion, postmortem studies mentioned above (Kovacs et al., 1999; Attems et al., 2005; Attems and Jellinger, 2006; Wilson et al., 2007, 2009) also state that the association between AD pathologic changes and OI largely reflects in the OB. Thus, it might be wiser to adopt a strategic regional analysis, rather than averaging the biomarker levels for the whole brain. Nevertheless, PET has so far not provided sufficient resolution measuring Aβ and/or tau deposition in the OB in humans, as stated by Risacher et al. (2017).

Furthermore, the prion-like hypothesis in AD is also worth noting concerning the olfactory impairment. Like the prion detected in olfactory epithelium of sporadic Creutzfeldt-Jakob disease (Tabaton et al., 2004), Aβ and tau also appeared in olfactory structures in AD and healthy subjects, including olfactory epithelium and OB (Kovacs et al., 1999; Wilson et al., 2007; Arnold et al., 2010; Brozzetti et al., 2020), both are susceptible to protein and enzyme modifications involved in AD pathogenesis (Dibattista et al., 2020). It was hypothesized that pathological modifications lead to the activation of protein accumulation in the OB after environmental insults, and then induces the propagation of the disease within the brain in a prion-like fashion by a templating process (Rey et al., 2018). Thus, OB was considered the entry site for this prion-like spreading in AD. Here, Aβ was proposed as an initiator for AD pathogenesis, while prion-like propagation of tauopathy dominated the process and might even independent of Aβ (Walker, 2018). Taken together, we believe these underlying pathologic development starting from olfactory neurons may contribute to the OI impairment, as memory dysfunction in AD and/or MCI was not enough to explain this deficit (Wilson et al., 2007; Reijs et al., 2017). Hence, the olfactory region-based association, again, between OI ability and Aβ, tau particularly is important. Recently, a non-invasive nasal brushing technique was used to collect the olfactory neuroepithelium (Brozzetti et al., 2020), from which neurodegeneration-associated proteins were detected, making correlating OI ability with Aβ and tau levels in peripheral olfactory system *in vivo* possible. This, certainly, may help clarify the relationship between the olfactory function and biomarkers of interest. A comprehensive search, a detailed subgroup analysis, and the appraisal of potential moderators for heterogeneity are the strengths of the study. In addition, active communication with the authors of the included studies and the comprehensive extraction of additional data provided our analysis with more power than the original publications offered.

However, several limitations of our study should also be noted. First, the available data did not allow us to correlate PET tau with OI ability or to identify associations within the AD group. In addition, other olfactory functions (olfactory threshold and discrimination) were not considered in this study. Thus, the inferences in the study cannot be extended to the above situation. Second, the sample sizes in general were modest but relatively small in the AD group and the CSF analysis, which reflects the limited amount of available data in the associated field and limits the power of detecting associations. Third, it was shown that the association was greater if the OI was defined by suggested cutoffs of abnormality, such as anosmia and normosmia (Vassilaki et al., 2017). Unfortunately, no such data could be obtained because only one study took the approach. Fourth, *t*-tau/Aβ_42_ or *p*-tau/Aβ_42_ might correlate with odor identification better than single CSF measures due to their improved ability for defining biomarker positivity, but the data was limited. Two studies reported *p*-tau/Aβ_42_, and the pooled results indicated a weak association (*r* = −0.17, *P* = 0.03). Fifth, it is advisable to take APOE ε4 allele into account when analyzing the associations between OI ability and amyloid-β and tau burden as the latter interacts with APOE ε4 allele. This has not been done as only a few studies adjusted APOE ε4 allele. Finally, the comprehensive data extraction was a double-edged sword, as it may have yielded results that deviate from the original results due to the recalculation and estimation of the data.

Future studies using PET tau imaging with larger sample sizes may help further clarify this issue. Concerning other olfactory functions, a recently published study (Lian et al., 2019) investigated the relationship between the threshold discrimination identification score and CSF Aβ and tau levels in AD patients with or without olfactory dysfunction (OD), finding that only *t*-tau levels were significantly lower in the AD-OD group, but the significant correlation disappeared after adjusting for age, sex, education, and disease duration. The same was true for the study by Doorduijn et al. (2020), which found no associations between AD biomarker levels and threshold discrimination identification. Another excluded study using odor percept identification performance with a homemade test to correlate with Aβ PET data also yielded a negative result (Dhilla Albers et al., 2016). These findings appear to be consistent with our meta-analysis results. Additionally, the *p*-tau/*t*-tau ratio has recently been shown to be related to olfaction in peripheral olfactory systems (Liu et al., 2018); thus, it might be interesting to examine the association between the *p*-tau/*t*-tau ratio and OI ability.

Here, we provide a thorough analysis on the negligible association between OI ability and Aβ and CSF tau burden, from the limitation of the OI test and Aβ measures themselves (both are lack of specificity) and the drawbacks of currently averaging the Aβ levels for the whole brain, to the possible association between the pathologic development (amyloid plaques and tauopathy) and OI impairment based on the prion-like hypothesis in AD. Specifically, we point out that the prion-like spreading Aβ, especially tau along the olfactory pathway (starting from OB) may contribute to the OI impairment, in parallel of memory dysfunction to some extent. This highlights a strategic regional analysis in the future, and a handful of other ideas, such as treating OI as a categorical variable (e.g., anosmia, normosmia), using *t*-tau/Aβ_42_ or *p*-tau/Aβ_42_, focusing on the APOE ε4 allele carriers, and most importantly measuring by PET tau imaging.

In summary, our meta-analysis suggests that OI impairment correlates marginally with Aβ PET data but not CSF Aβ and more weakly correlates with CSF *t*-tau but not *p*-tau. These findings may disappoint those who intend to use OI ability alone for the early detection of AD. Nevertheless, PET tau might be more strongly associated with OI impairment; however, more studies are needed to clarify this association. Importantly, our results should not be regarded as a rationale for denying the value of olfactory testing in AD research. OI test may have limited value predicting amyloid and tau status when used alone, yet it is possible that an enhanced association between the two may be yielded when combined with other biologic markers discussed above (e.g., focusing on APOE ε4 carriers using tau PET imaging with olfactory region-based analysis), and it is still valuable in predicting cognitive decline and progression from MCI to AD dementia. In fact, as a low-cost, non-invasive method of evaluating olfactory function, the assessment of OI ability, combined with global cognitive testing, has the potential to help clinicians identify persons who rarely transition to dementia (Devanand et al., 2019), thus helping practitioners decide whether to apply further diagnostic investigations, such as PET scans, which help reduce the burden and cost of clinical AD trials and as the first diagnostic tau radiotracer for use with PET was approved by the US Food and Drug Administration, further research is possible and warranted.

## Data Availability Statement

The original contributions presented in the study are included in the article/supplementary material, further inquiries can be directed to the corresponding author/s.

## Author Contributions

LT contributed to the study design, data analysis, and interpretation and drafted the manuscript. XL contributed to data analysis and interpretation and critical revision of the manuscript. ZF and MZ contributed to data analysis and revision of the manuscript. XY and HW contributed to the design of this study, interpretation of data, and critical revision of the manuscript and had primary responsibility for the final content. All authors read and approved the final manuscript. All authors contributed to the article and approved the submitted version.

## Conflict of Interest

The authors declare that the research was conducted in the absence of any commercial or financial relationships that could be construed as a potential conflict of interest.

## References

[B1] Alzheimer's Association (2020). Alzheimer's disease facts and figures. Alzheimer's Dement. 16, 391–460. 10.1002/alz.12068

[B2] Arnold S. E. Lee E. B. Moberg P. J. Stutzbach L. Kazi H. Han L.-Y.. (2010). Olfactory epithelium amyloid-beta and paired helical filament-tau pathology in Alzheimer disease. Ann. Neurol. 67, 462–469. 10.1002/ana.21910 20437581 PMC2864948

[B3] Attems J. Jellinger K. A. (2006). Olfactory tau pathology in Alzheimer disease and mild cognitive impairment. Clin. Neuropathol. 25, 265–271. 17140156

[B4] Attems J. Lintner F. Jellinger K. A. (2005). Olfactory involvement in aging and Alzheimer's disease: an autopsy study. J. Alzheimers Dis. 7, 149–157; discussion 173–180. 10.3233/JAD-2005-7208 15851853

[B5] Bahar-Fuchs A. Chételat G. Villemagne V. L. Moss S. Pike K. Masters C. L.. (2010). Olfactory deficits and amyloid-β burden in Alzheimer's disease, mild cognitive impairment, and healthy aging: a PiB PET study. J. Alzheimers Dis. 22, 1081–1087. 10.3233/JAD-2010-100696 20930316

[B6] Brier M. R. Gordon B. Friedrichsen K. McCarthy J. Stern A. Christensen J.. (2016). Tau and Aβ imaging, CSF measures, and cognition in Alzheimer's disease. Sci. Transl. Med. 8:338ra66. 10.1126/scitranslmed.aaf2362 27169802 PMC5267531

[B7] Brozzetti L. Sacchetto L. Cecchini M. P. Avesani A. Perra D. Bongianni M.. (2020). Neurodegeneration-associated proteins in human olfactory neurons collected by nasal brushing. Front. Neurosci. 14:145. 10.3389/fnins.2020.00145 32194369 PMC7066258

[B8] Devanand D. P. Lee S. Luchsinger J. A. Andrews H. Goldberg T. Huey E. D.. (2019). Intact global cognitive and olfactory ability predicts lack of transition to dementia. Alzheimer's Dement. 15:P1535. 10.1016/j.jalz.2019.08.112 31676234 PMC7007828

[B9] Devanand D. P. Lee S. Manly J. Andrews H. Schupf N. Doty R. L.. (2015). Olfactory deficits predict cognitive decline and Alzheimer dementia in an urban community. Neurology 84, 182–189. 10.1212/WNL.0000000000001132 25471394 PMC4336090

[B10] Dhilla Albers A. Asafu-Adjei J. Delaney M. K. Kelly K. E. Gomez-Isla T. Blacker D.. (2016). Episodic memory of odors stratifies Alzheimer biomarkers in normal elderly. Ann. Neurol. 80, 846–857. 10.1002/ana.24792 27696605 PMC5177522

[B11] Dibattista M. Pifferi S. Menini A. Reisert J. (2020). Alzheimer's disease: what can we learn from the peripheral olfactory system? Front. Neurosci. 14:440. 10.3389/fnins.2020.00440 32508565 PMC7248389

[B12] Dintica C. S. Marseglia A. Rizzuto D. Wang R. Seubert J. Arfanakis K.. (2019). Impaired olfaction is associated with cognitive decline and neurodegeneration in the brain. Neurology 92:e700–9. 10.1212/WNL.0000000000006919 30651382 PMC6382360

[B13] Doorduijn A. S. de van der Schueren M. A. E. van de Rest O. de Leeuw F. A. Fieldhouse J. L. P. Kester M. I.. (2020). Olfactory and gustatory functioning and food preferences of patients with Alzheimer's disease and mild cognitive impairment compared to controls: the NUDAD project. J. Neurol. 267, 144–152. 10.1007/s00415-019-09561-0 31595376 PMC6954901

[B14] Doty R. L. Kamath V. (2014). The influences of age on olfaction: a review. Front. Psychol. 5:20. 10.3389/fpsyg.2014.00020 24570664 PMC3916729

[B15] Doty R. L. Shaman P. Applebaum S. L. Giberson R. Siksorski L. Rosenberg L. (1984). Smell identification ability: changes with age. Science 226, 1441–1443. 10.1126/science.6505700 6505700

[B16] Franks K. H. Chuah M. I. King A. E. Vickers J. C. (2015). Connectivity of pathology: the olfactory system as a model for network-driven mechanisms of alzheimer's disease pathogenesis. Front. Aging. Neurosci. 7:234. 10.3389/fnagi.2015.00234 26696886 PMC4678206

[B17] Growdon M. E. Schultz A. P. Dagley A. S. Amariglio R. E. Hedden T. Rentz D. M.. (2015). Odor identification and Alzheimer disease biomarkers in clinically normal elderly. Neurology 84, 2153–2160. 10.1212/WNL.0000000000001614 25934852 PMC4451046

[B18] Hoyles K. Sharma J. C. (2013). Olfactory loss as a supporting feature in the diagnosis of Parkinson's disease: a pragmatic approach. J. Neurol. 260, 2951–2958. 10.1007/s00415-013-6848-8 23377435

[B19] Ingelsson M. Fukumoto H. Newell K. L. Growdon J. H. Hedley-Whyte E. T. Frosch M. P.. (2004). Early Abeta accumulation and progressive synaptic loss, gliosis, and tangle formation in AD brain. Neurology 62, 925–931. 10.1212/01.WNL.0000115115.98960.37 15037694

[B20] Jack C. R. Bennett D. A. Blennow K. Carrillo M. C. Dunn B. Haeberlein S. B.. (2018). NIA-AA research framework: toward a biological definition of Alzheimer's disease. Alzheimers Dement. 14, 535–562. 10.1016/j.jalz.2018.02.018 29653606 PMC5958625

[B21] Jack C. R. Wiste H. J. Therneau T. M. Weigand S. D. Knopman D. S. Mielke M. M.. (2019). Associations of amyloid, tau, and neurodegeneration biomarker profiles with rates of memory decline among individuals without dementia. JAMA 321, 2316–2325. 10.1001/jama.2019.7437 31211344 PMC6582267

[B22] Jansen W. J. Ossenkoppele R. Knol D. L. Tijms B. M. Scheltens P. Verhey F. R. J. (2015). Prevalence of cerebral amyloid pathology in persons without dementia: a meta-analysis. JAMA 313, 1924–1938. 10.1001/jama.2015.4668 25988462 PMC4486209

[B23] Jung H. J. Shin I.-S. Lee J.-E. (2019). Olfactory function in mild cognitive impairment and Alzheimer's disease: a meta-analysis. Laryngoscope 129, 362–369. 10.1002/lary.27399 30565695

[B24] Kamath V. Crawford J. DuBois S. Nucifora F. C. Jr. Nestadt G. Sawa A.. (2019). Contributions of olfactory and neuropsychological assessment to the diagnosis of first-episode schizophrenia. Neuropsychology 33, 203–211. 10.1037/neu0000502 30475048 PMC10123949

[B25] Kametani F. Hasegawa M. (2018). Reconsideration of amyloid hypothesis and tau hypothesis in alzheimer's disease. Front. Neurosci. 12:25. 10.3389/fnins.2018.00025 29440986 PMC5797629

[B26] Kim M. J. Moon S. Oh B.-C. Jung D. Choi K. Park Y. J. (2019). Association between diethylhexyl phthalate exposure and thyroid function: a meta-analysis. Thyroid 29, 183–192. 10.1089/thy.2018.0051 30588877 PMC6488044

[B27] Körtvélyessy P. Gukasjan A. Sweeney-Reed C. M. Heinze H.-J. Thurner L. Bittner D. M. (2015). Progranulin and Amyloid-β levels: relationship to neuropsychology in frontotemporal and alzheimer's disease. J. Alzheimers Dis. 46, 375–380. 10.3233/JAD-150069 25777512

[B28] Kouzuki M. Suzuki T. Nagano M. Nakamura S. Katsumata Y. Takamura A.. (2018). Comparison of olfactory and gustatory disorders in Alzheimer's disease. Neurol. Sci. 39, 321–328. 10.1007/s10072-017-3187-z 29128987

[B29] Kovacs T. Cairns N. J. Lantos P. L. (1999). beta-amyloid deposition and neurofibrillary tangle formation in the olfactory bulb in ageing and Alzheimer's disease. Neuropathol. Appl. Neurobiol. 25, 481–491. 10.1046/j.1365-2990.1999.00208.x 10632898

[B30] Kreisl W. C. Jin P. Lee S. Dayan E. R. Vallabhajosula S. Pelton G.. (2018). Odor identification ability predicts PET amyloid status and memory decline in older adults. J. Alzheimers Dis. 62, 1759–1766. 10.3233/JAD-170960 29614678 PMC6760657

[B31] Lafaille-Magnan M.-E. Poirier J. Etienne P. Tremblay-Mercier J. Frenette J. Rosa-Neto P.. (2017). Odor identification as a biomarker of preclinical AD in older adults at risk. Neurology 89, 327–335. 10.1212/WNL.0000000000004159 28659431 PMC5574678

[B32] Leuzy A. Chiotis K. Lemoine L. Gillberg P.-G. Almkvist O. Rodriguez-Vieitez E.. (2019). Tau PET imaging in neurodegenerative tauopathies-still a challenge. Mol. Psychiatry 24, 1112–1134. 10.1038/s41380-018-0342-8 30635637 PMC6756230

[B33] Lian T.-H. Zhu W.-L. Li S.-W. Liu Y.-O. Guo P. Zuo L.-J.. (2019). Clinical, structural, and neuropathological features of olfactory dysfunction in patients with alzheimer's disease. J. Alzheimer's Dis. 70, 413–423. 10.3233/JAD-181217 31177212

[B34] Liu Z. Kameshima N. Nanjo T. Shiino A. Kato T. Shimizu S.. (2018). Development of a high-sensitivity method for the measurement of human nasal Aβ 42, Tau, and phosphorylated Tau. J. Alzheimer's Dis. 62, 737–744. 10.3233/JAD-170962 29480194 PMC5836401

[B35] Mahlknecht P. Iranzo A. Hogl B. Frauscher B. Muller C. Santamaria J.. (2015). Olfactory dysfunction predicts early transition to a Lewy body disease in idiopathic RBD. Neurology 84, 654–658. 10.1212/WNL.0000000000001265 25609758

[B36] Marin C. Vilas D. Langdon C. Alobid I. López-Chacón M. Haehner A.. (2018). Olfactory dysfunction in neurodegenerative diseases. Curr. Allergy Asthma Rep. 18:42. 10.1007/s11882-018-0796-4 29904888

[B37] Mattsson N. Schöll M. Strandberg O. Smith R. Palmqvist S. Insel P. S.. (2017). 18F-AV-1451 and CSF T-tau and P-tau as biomarkers in Alzheimer's disease. EMBO Mol. Med. 9, 1212–1223. 10.15252/emmm.201707809 28743782 PMC5582410

[B38] Meyer P.-F. Binette A. P. Gonneaud J. Breitner J. C. S. Villeneuve S. (2020). Characterization of alzheimer disease biomarker discrepancies using cerebrospinal fluid phosphorylated tau and AV1451 positron emission tomography. JAMA Neurol. 77, 508–516. 10.1001/jamaneurol.2019.4749 31961372 PMC6990861

[B39] Moher D. Shamseer L. Clarke M. Ghersi D. Liberati A. Petticrew M.. (2015). Preferred reporting items for systematic review and meta-analysis protocols (PRISMA-P) 2015 statement. Syst. Rev. 4:1. 10.1186/2046-4053-4-1 25554246 PMC4320440

[B40] Nag S. Yu L. VanderHorst V. G. Schneider J. A. Bennett D. A. Buchman A. S.. (2019). Neocortical lewy bodies are associated with impaired odor identification in community-dwelling elders without clinical PD. J. Neurol. 266, 3108–3118. 10.1007/s00415-019-09540-5 31535271 PMC6851442

[B41] Reijs B. L. R. Ramakers I. H. G. B. Elias-Sonnenschein L. Teunissen C. E. Koel-Simmelink M. Tsolaki M.. (2017). Relation of odor identification with alzheimer's disease markers in cerebrospinal fluid and cognition. J. Alzheimers Dis. 60, 1025–1034. 10.3233/JAD-170564 28984603

[B42] Rey N. L. Wesson D. W. Brundin P. (2018). The olfactory bulb as the entry site for prion-like propagation in neurodegenerative diseases. Neurobiol. Dis. 109, 226–248. 10.1016/j.nbd.2016.12.013 28011307 PMC5972535

[B43] Risacher S. L. Tallman E. F. West J. D. Yoder K. K. Hutchins G. D. Fletcher J. W. (2017). Olfactory identification in subjective cognitive decline and mild cognitive impairment: association with tau but not amyloid positron emission tomography. Alzheimers Dement. 9, 57–66. 10.1016/j.dadm.2017.09.001 PMC5675709 29159268

[B44] Roalf D. R. Moberg M. J. Turetsky B. I. Brennan L. Kabadi S. Wolk D. A.. (2017). A quantitative meta-analysis of olfactory dysfunction in mild cognitive impairment. J. Neurol. Neurosurg. Psychiatr. 88, 226–232. 10.1136/jnnp-2016-314638 28039318 PMC5350628

[B45] Roberts R. O. Christianson T. J. H. Kremers W. K. Mielke M. M. Machulda M. M. Vassilaki M.. (2016). Association between olfactory dysfunction and amnestic mild cognitive impairment and Alzheimer disease dementia. JAMA Neurol. 73, 93–101. 10.1001/jamaneurol.2015.2952 26569387 PMC4710557

[B46] Serrano-Pozo A. Mielke M. L. Gómez-Isla T. Betensky R. A. Growdon J. H. Frosch M. P.. (2011). Reactive Glia not only associates with plaques but also parallels tangles in alzheimer's disease. Am. J. Pathol. 179, 1373–1384. 10.1016/j.ajpath.2011.05.047 21777559 PMC3157187

[B47] Shankar G. M. Li S. Mehta T. H. Garcia-Munoz A. Shepardson N. E. Smith I.. (2008). Amyloid β-protein dimers isolated directly from alzheimer brains impair synaptic plasticity and memory. Nat. Med. 14, 837–842. 10.1038/nm1782 18568035 PMC2772133

[B48] Sterne J. A. C. Sutton A. J. Ioannidis J. P. A. Terrin N. Jones D. R. Lau J.. (2011). Recommendations for examining and interpreting funnel plot asymmetry in meta-analyses of randomised controlled trials. BMJ 343:d4002. 10.1136/bmj.d4002 21784880

[B49] Tabaton M. Monaco S. Cordone M. P. Colucci M. Giaccone G. Tagliavini F.. (2004). Prion deposition in olfactory biopsy of sporadic Creutzfeldt-Jakob disease. Ann. Neurol. 55, 294–296. 10.1002/ana.20038 14755736

[B50] Thomann P. A. Dos Santos V. Toro P. Schönknecht P. Essig M. Schröder J. (2009). Reduced olfactory bulb and tract volume in early Alzheimer's disease–a MRI study. Neurobiol. Aging 30, 838–841. 10.1016/j.neurobiolaging.2007.08.001 17875348

[B51] Tu L.-H. Wang H.-L. Yu X. (2020). Olfactory dysfunction and common neurocognitive disorder: an update of research progression. Chinese J. Psychiatr. 53, 155–158. 10.3760/cma.j.cn113661-20190426-00151

[B52] Vasavada M. M. Martinez B. Wang J. Eslinger P. J. Gill D. J. Sun X. (2017). Central olfactory dysfunction in alzheimer's disease and mild cognitive impairment: a functional MRI study. J. Alzheimers Dis. 59, 359–368. 10.3233/JAD-170310 28671131

[B53] Vasavada M. M. Wang J. Eslinger P. J. Gill D. J. Sun X. Karunanayaka P.. (2015). Olfactory cortex degeneration in Alzheimer's disease and mild cognitive impairment. J. Alzheimers Dis. 45, 947–958. 10.3233/JAD-141947 25633674

[B54] Vassilaki M. Christianson T. J. Mielke M. M. Geda Y. E. Kremers W. K. Machulda M. M.. (2017). Neuroimaging biomarkers and impaired olfaction in cognitively normal individuals. Ann. Neurol. 81, 871–882. 10.1002/ana.24960 28543731 PMC5517772

[B55] Villemagne V. L. Dor,é V. Burnham S. C. Masters C. L. Rowe C. C. (2018). Imaging tau and amyloid-β proteinopathies in Alzheimer disease and other conditions. Nat. Rev. Neurol. 14, 225–236. 10.1038/nrneurol.2018.9 29449700

[B56] Waldton S. (1974). Clinical observations of impaired cranial nerve function in senile dementia. Acta Psychiatr. Scand. 50, 539–547. 10.1111/j.1600-0447.1974.tb09714.x 4460686

[B57] Walker L. C. (2018). Prion-like mechanisms in Alzheimer disease. Handb. Clin. Neurol. 153, 303–319. 10.1016/B978-0-444-63945-5.00016-7 29887142 PMC6375694

[B58] Walsh D. M. Klyubin I. Fadeeva J. V. Cullen W. K. Anwyl R. Wolfe M. S.. (2002). Naturally secreted oligomers of amyloid β protein potently inhibit hippocampal long-term potentiation *in vivo*. Nature 416, 535–539. 10.1038/416535a 11932745

[B59] Whiting P. F. Rutjes A. W. S. Westwood M. E. Mallett S. Deeks J. J. Reitsma J. B.. (2011). QUADAS-2: a revised tool for the quality assessment of diagnostic accuracy studies. Ann. Intern. Med. 155, 529–536. 10.7326/0003-4819-155-8-201110180-00009 22007046

[B60] Wilson R. S. Arnold S. E. Schneider J. A. Boyle P. A. Buchman A. S. Bennett D. A. (2009). Olfactory impairment in presymptomatic Alzheimer's disease. Ann. N. Y. Acad. Sci. 1170, 730–735. 10.1111/j.1749-6632.2009.04013.x 19686220 PMC2857767

[B61] Wilson R. S. Arnold S. E. Schneider J. A. Tang Y. Bennett D. A. (2007). The relationship between cerebral Alzheimer's disease pathology and odour identification in old age. J. Neurol. Neurosurg. Psychiatr. 78, 30–35. 10.1136/jnnp.2006.099721 17012338 PMC2117790

[B62] Wu X. Geng Z. Zhou S. Bai T. Wei L. Ji G.-J.. (2019). Brain structural correlates of odor identification in mild cognitive impairment and alzheimer's disease revealed by magnetic resonance imaging and a chinese olfactory identification test. Front. Neurosci. 13:842. 10.3389/fnins.2019.00842 31474819 PMC6702423

[B63] Yu J.-T. Li J.-Q. Suckling J. Feng L. Pan A. Wang Y.-J.. (2019). Frequency and longitudinal clinical outcomes of Alzheimer's AT(N) biomarker profiles: a longitudinal study. Alzheimers Dement 15, 1208–1217. 10.1016/j.jalz.2019.05.006 31399333

